# Genomic Insights into Vector–Pathogen Adaptation in *Haemaphysalis longicornis* and *Rhipicephalus microplus*

**DOI:** 10.3390/pathogens14040306

**Published:** 2025-03-23

**Authors:** Jin Liu, An Zhou, Qi Liu, Yang Gao, Shuhua Xu, Yan Lu

**Affiliations:** 1 State Key Laboratory of Genetic Engineering, Collaborative Innovation Center of Genetics and Development, School of Life Sciences, Fudan University, Shanghai 200438, China; 22210700035@m.fudan.edu.cn (J.L.); gaoyang@fudan.edu.cn (Y.G.); xushua@fudan.edu.cn (S.X.); 2 Center for Evolutionary Biology, Ministry of Education Key Laboratory of Contemporary Anthropology, Fudan University, Shanghai 200438, China; 18375373919@163.com; 3 Department of Liver Surgery and Transplantation Liver Cancer Institute, Zhongshan Hospital, Fudan University, Shanghai 201203, China; liuqi_@fudan.edu.cn

**Keywords:** *Haemaphysalis longicornis*, *Rhipicephalus microplus*, tick-borne disease, vector–pathogen adaptation

## Abstract

As crucial vectors that transmit pathogens to humans and livestock, ticks pose substantial global health threats and economic burdens. We analyzed 328 tick genomes to explore the population’s genetic structure and the adaptive evolution of *H. longicornis* and *R. microplus,* two tick species with distinct life cycle characteristics. We observed distinct genetic structures in *H. longicornis* and *R. microplus*. Gene flow estimation revealed a closer genetic connection in *R. microplus* than *H. longicornis*, which was facilitated by geographical proximity. Notably, we identified a set of candidate genes associated with possible adaptations. Specifically, the immune-related gene *DUOX* and the iron transport gene *ACO1* showed significant signals of natural selection in *R. microplus*. Similarly, *H. longicornis* exhibited selection in pyridoxal-phosphate-dependent enzyme genes associated with heme synthesis. Moreover, we observed significant correlations between the abundance of pathogens, such as *Rickettsia* and *Francisella,* and specific tick genotypes, which highlights the role of *R. microplus* in maintaining these pathogens and its adaptations that influence immune responses and iron metabolism, suggesting potential coevolution between vectors and pathogens. Our study highlights the vital genes involved in tick blood feeding and immunity, and it provides insights into the coevolution of ticks and tick-borne pathogens.

## 1. Introduction

Ticks, obligate bloodsucking, nonpermanent ectoparasitic arthropods, are one of the most important vectors for globally transmitting pathogens to humans and domestic animals [1,2]. Ticks include 702 species of hard ticks, 193 species of soft ticks, and 1 species of *Nuttalliella* [3]. Furthermore, ticks serve as a conduit for an extensive array of pathogenic microorganisms, surpassing the diversity transmitted by any other cohort of arthropod vectors. They often carry more than one pathogen and cause a series of tick-borne diseases (TBDs), including Lyme borreliosis, anaplasmosis, babesiosis, and ehrlichiosis [1,3,4,5,6]. Lyme disease cases treated in the United States number 476,000, and there are an estimated 700,000 human cases per year in the United States of America plus Europe [7,8]. Lyme borreliosis primarily spreads through multiple species of hard ticks, and each year, approximately 255,000 individuals worldwide fall ill due to this ailment, which is predominantly concentrated in Europe and North America, and approximately 30,000 cases are reported annually in northern China [9,10]. The economic repercussions of tick-borne illnesses are notable and consistently escalating [11,12,13,14]. These diseases affect nearly 80% of the global cattle population, resulting in estimated global costs of tick and tickborne disease ranging from USD 13.9 to 18.7 billion [15]. Thus, TBDs pose a grave threat to global public health and impose substantial economic burdens on the livestock industry.

The Asian long-horned tick, *H. longicornis*, has a remarkable capacity to endure a wide range of temperatures, from a developmental threshold of approximately 12 °C to a critical limit of nearly 40 °C [16]. *H. longicornis,* documented in ten countries, has a versatile host range and is a vector for numerous pathogens. At least 30 human pathogens have been associated with *H. longicornis*, including seven species of spotted fever group *rickettsiae*, species in the family of *Anaplasmataceae*, four genospecies in the complex *Borrelia burgdorferi* sensu lato, two *Babesia* species, six species of virus including severe fever with thrombocytopenia syndrome virus, Jingmen tick virus, bocavirus, Nairobi sheep disease virus, lymphocytic choriomeningitis virus, tick-borne encephalitis virus, and *Francisella*, *Bartonella*, *Coxiella*, and *Toxoplasma*, which were mainly reported in eastern Asia [17]. In China, *H. longicornis* is the most common tick species, with reports identifying at least 15 associated agents, 10 of which have potential pathogenicity to human health [18,19]. As a three-host invasive tick, *H. longicornis* has demonstrated rapid territorial expansion and robust proliferation within established habitats. Interestingly, this species has shown no discernible geographic structuring within mainland China, highlighting its ecological plasticity and dynamic population structure [20]. 

In contrast, the cattle tick, *R. microplus*, a one-host tick, exhibits a distinctive geographical dispersal strategy, resulting in significant economic implications for the cattle industry, particularly in tropical and subtropical regions across the globe. Moreover, *R. microplus* is a vector for the pathogens responsible for babesiosis (*Babesia bovis* and *B. bigemina*) and anaplasmosis (*Anaplasma marginale*) [21,22,23]. *R. microplus* has a more distinct population structure in China. A comprehensive comparative analysis of these two tick species in their native range, including analyzing their genetic structures and genomic attributes, influenced by their respective host interaction strategies, can yield valuable insights. Moreover, the difference in gene expression in their different tissues and development stages should be explored to provide a deeper understanding of their individual mechanisms [20].

Understanding the functional impact of pathogen colonization and transmission within tick carriers is imperative for developing innovative strategies to control ticks and tick-borne pathogens using targeted protein interventions [24]. The manipulation of host defenses by ticks is one of the elements that determines the branch of pathogen that a tick successfully transmits. The interaction of ticks and tick-borne pathogens at the host skin interface is critical to establishing an environment conducive to pathogen transmission and development. Ticks can promote blood feeding by inhibiting salivary secretion or regulating host defense [25]. There are several ways to regulate host defense, mainly reducing the host’s perception of pain through immunomodulatory responses in tick saliva. The saliva of some tick species contains kininase and amine (histamine)-binding lipid carrier proteins, which reduce pain and itch responses [26,27]. This is followed by tick inhibition of host hemostasis through the secretion of salivary vasodilators, platelet aggregation inhibitors, and molecules that delay or inhibit components of the clotting cascade and further regulate host wound healing inflammation, inhibit host immune response, and affect pathogen transmission [28,29,30,31]. In addition, different tick species may have multiple immune defense strategies to counteract the host’s major categories of defense regulation, which will be more conducive to pathogen transmission, establishment, and transmission environment [32,33].

The interaction between ticks and pathogens is very complex, including conflict and cooperation, and involves many metabolic pathways, such as carbohydrates, proteins, lipids, and redox pathways. However, both ticks and pathogens benefit from these action mechanisms for survival [34]. Ticks can participate in phagocytosis of different microorganisms through cellular immunity mediated by blood cells [35]. Iron metabolism in ticks may have a role in microbial infection, which is central to host–pathogen interactions [36]. Ticks and pathogens have coevolved mechanisms for acquiring iron from each other, and this ’nutritional immunity’ is essential to maintain balance between ticks and pathogens [37]. Nevertheless, tick-borne pathogen infections induce transcriptional reprogramming, which affects several metabolic pathways in ticks, promoting infection, reproduction, and transmission [38]. They also help the tick complete its life cycle in harsh environments, improving its survival performance [39,40]. Therefore, in some specific cases, vector tolerance to pathogen infection is also a favorable feature of the coevolution of the two [41]. Notably, the most prevalent bacterial genera in ticks are *Coxiella* (60.5%) and *Rickettsia* (55.6%), with a broader distribution across tick species than any other genus [42]. Within tick organs, *Rickettsia* regulates the tick genome, manipulating robust anti-oxidant mechanisms to evade increases in reactive oxygen species (ROS) through a range of selenoproteins and non-selenoproteins (e.g., SOD, catalase, and glutathione reductase) [24]. These findings reveal the coevolution mechanism of ticks and pathogens, and further understanding of tick adaptation to survival, transmission, and tick–pathogen interactions provides an important opportunity to identify new therapeutic targets for tick-borne disease prevention and control [34].

Single-nucleotide polymorphisms (SNPs) are the most fundamental form of DNA variation among individuals and can induce alterations in encoded amino acids (non-synonymous), remain silent with no effect on the amino acid sequence (synonymous), or manifest in non-coding regions [43]. These variations have considerable implications, revealing genomic evolutionary processes and inter-individual genetic disparities while also facilitating an in-depth analysis of mechanisms underlying diseases such as diabetes, bipolar disorder, and hypertension [43,44,45,46,47,48]. However, little is known about how SNPs affect the biological functions of ticks and the interactions between tick pathogens and hosts.

Since little is known about the genetic variations of ticks and the interactions between tick-borne pathogens and the genetic background of ticks, we generated high-quality SNP data from the 161 *H. longicornis* and 140 *R. microplus* genomes. Then, we performed a genetic analysis to examine the evolutionary history and local adaptation of the two tick species, with a particular focus on blood digestion and immune defense mechanisms, given that these species have distinct host-associated life cycles. Moreover, we carried out an association study on the potential interactions between ticks and pathogens to explore the coevolution between pathogens and tick genomes. Our study highlighted the genetic variations related to host adaptability traits, thereby enriching our understanding of the intricate genomic mechanisms driving tick adaptation to their respective hosts and dynamic environments.

## 2. Materials and Methods

### 2.1. Data Collection and Alignment

The re-sequenced data of 177 *H. longicornis* and 151 *R. microplus* were downloaded from the Genome Sequence Archive database (GSA: PRJCA002242) [20]. Reads of the 328 tick samples were aligned to the corresponding reference genome (ASM1333976v2 and ASM1333972v1, respectively) using Burrows–Wheeler Aligner (BWA) (version 0.7.17) [49]. The SNP callings were performed using the Genome Analysis Toolkit (GATK) (version 4.2.4.1) [50]. Because of unknown sampling locations and a low genomic mapping rate (<50%), 15 *H. longicornis* and 11 *R. microplus* were filtered out. Finally, 161 samples of *H. longicornis* and 140 samples of *R. microplus* were retained for further analysis.

### 2.2. Alignment, Variant Calling, and Annotation

Sequencing reads were filtered by removing adaptors and low-quality bases using fastp (version 0.19.3) [51]. This study mapped only qualified pair-end reads to the reference genome (ASM1333976v2 and ASM1333972v1) using BWA-MEM with the -M parameter [52]. The resulting BAM files were marked as duplicates and locally realigned around indels using GATK (version 4.2.4.1) [50]. Because no whole-genome SNP dataset was available for the Base Quality Score Recalibrator (BQSR), we followed the approach recommended on the GTAK website for non-human data. An initial round of variant calling was performed using GATK HaplotypeCaller, and a joint genotyping step for comprehensive variation union was performed on the gVCF files with GATK GenotypeGVCFs. A hard filter was subsequently performed to filter the variants using GATK tools, and the parameters were set by default (for SNPs: QD < 2.0, FS > 60.0, MQ < 40.0, MQRankSum < 12.5, ReadPosRankSum < 8.0). The variants that passed the hard filter were used as a true positive set of variant sites for BQSR. Variant calling was repeated for the recalibrated BAM files, and then, the whole cohort was re-genotyped using GATK. We excluded sites with missing rates > 10% and MAF (minor allele frequency) < 5% in all samples using VCFtools (version 0.1.16) [53]. Functional annotation of SNPs was performed according to the tick genome using the software ANNOVAR (version 2020-6-7) [54].

### 2.3. Genetic Structure Analyses

First, we performed population structure analysis by filtering out the variants with a missing rate > 10% and MAF < 5% for all samples, and the LD was eliminated by using VCFtools and down-sampled according to the physical interval of no less than 5 kb between any two variants. Moreover, PCA was performed using PLINK (version 1.9) with the thinning SNP datasets [55]. We used the maximum likelihood tree-based approach implemented in IQtree (version 1.6.12) with the GTR+F+R6+ASC model [56]. The population genetic structure was inferred using ADMIXTIRE software (version 1.3.0) [57]. Ten independent runs for each K = 2–10 were carried out, and the process was repeated 10 times with different random seeds. The lowest cross-validation error (CV) value was found in *R. microplus* for K = 2, increasing for each value of K. We used AncestryPainter (version 1.1) to describe the summation and graphical representation of ADMIXTIRE outputs [58].

VCFtools (version 0.1.16) was used to calculate the nucleotide diversity (π) and fixation index (F_ST_) based on all autosomal high-quality SNPs with a 10 kb window and a step size of 5 kb for every subpopulation [53]. We also calculated Tajima’s *D* in 10-kb non-overlapping windows across each autosome for each population. Tajima’s *D* values for each population were calculated using the same sliding window approach with an in-house script. Finally, LD decay was estimated using the PopLDdecay tool (version 3.42) that calculates the genotype correlation coefficient (r^2^) for pairs of all autosomal unphased SNPs at a maximum distance range of 300 kb [59]. For each subpopulation of *H. longicornis* and *R. microplus*, the LD decay was calculated separately and defined by the SubPop option. The LD decay was measured as the chromosomal distance at which the average pairwise r^2^ decreased to half its maximum value.

We used TreeMix (version 1.13) to estimate the flow of genes across each species [60]. Additionally, we used the estimated effective migration surface (EEMS) to estimate genetic migration patterns in specific geographic areas and compare geographic and genetic distances between different communities by calculating a genetic dissimilarity matrix and assigning geographic coordinates [61]. Finally, the R script provided by EEMS was used to visualize the result in space.

### 2.4. Detection of Genome-Wide Selection Signals

Multiple tests were used to investigate selection, including cross-population (F_ST_ and XP-EHH) and within-population methods (iHS and Tajima’s *D*) in *H. longicornis* and *R. microplus*. To identify the candidate regions of selection, we calculated F_ST_ in a 10 kb sliding window with a step size of 5 kb using VCFtools, and an empirical threshold of 1% was chosen as the outlier window. Statistical phasing was performed using Beagle (v. 5.4) [62]. Then, all the phased bi-allelic variants were used to calculate the integrated haplotype scores (iHS) and cross-population extended haplotype homozygosity (XP-EHH) using Selscan (v. 1.3.0) for each chromosome separately [63]. We also used the norm (v. 1.3.0) distributed with Selscan to normalize all Selscan outputs across all chromosomes. Moreover, the *p*-value of iHS and XP-EHH scores were calculated for 10 kb non-overlapping windows along each chromosome. Windows with a top 1% *p*-value were considered the selection signal. The same sliding windows with two or more significant SNPs were identified as selected genomic regions. Moreover, PBscan was applied to compute the population branching statistic (PBS) [64].

### 2.5. Association of Microbial Composition with Tick SNPs

Tick sequences were filtered using SAMtools (version 0.9.24) after mapping the reads of 301 specimens to tick genomes by BWA (version: 0.7.17), and all unmapped reads were retained for subsequent analysis [52,65]. Taxonomic classification was performed by aligning the filtered reads to the NR database using DIAMOND (version 0.9.24, parameters: -f 102 -top 10) [66]. To estimate the relative abundances of different microbial species, we extracted all taxonomic IDs according to the NCBI taxdump ftp://ftp.ncbi.nlm.nih.gov/pub/taxonomy/taxdmp.zip (accessed on 12 August 2023) (*Rickettsia*: TaxID780, *Anaplasma*: TaxID768, *Ehrlichia*: TaxID 943, *Borrelia*: TaxID138, *Babesia*: TaxID 5864, *Theileria*: TaxID 5873, *Francisella*: TaxID262, *Bartonella*: TaxID 773, *Coxiella*: TaxID 776, *Hepatozoon*: TaxID 75741, *Toxoplasma*: TaxID 5810, and *Candidatus Neoehrlichia*: TaxID 467749). Sequence similarity (>70%) was used as the threshold to screen the alignment results. The union set of the metagenome species that had significantly different abundance between different regions in two ticks (*p* < 0.05, FDR < 10%, nonparametric two-sided Wilcoxon rank-sum test; Tables S8–S11) was retained for the correlation analysis. The union set of the host SNPs with the top 0.01 F_ST_ between SCC and SEC, SCC and SWC, SEC and SWC, and domestic and overseas populations of the two ticks was selected for the correlation analysis. To decrease the influence of LD, we filtered these SNPs using the tag SNP tagger from Paul de Bakker’s Tagger tag SNP selection algorithm in Haploview [67]. Thus, 28,133 filtered SNPs in *R. microplus* and 4229 in *H. longicornis* were recruited for the correlation analysis. Normalized abundance values of metagenome species were treated as quantitative traits for the rank-based Spearman correlation analysis to identify the correlation between the genotype of each SNP. The union set of the host SNPs with a strong selection signal in the two tick species was selected for the correlation analysis. Samples of a single ethnic group in *R. microplus* and *H. longicornis* were used to calculate the *p*-value of a single group using the Spearman correlation test, followed by a meta-analysis to produce the meta-*p*-value and the adjusted meta-*p*-value. The results were determined to be significant if meta-*p* < 0.05, adjusted meta-*p* < 0.05, and FDR < 10%.

### 2.6. Functional Enrichments and Differential Expression Analysis of Genes

The functional enrichments of the protein-coding genes were performed by using multiple gene annotation software, including eggNOG-mapper http://eggnog-mapper.embl.de/ (accessed on 9 January 2023), BlastKOALA in KEGG https://www.kegg.jp/blastkoala/ (accessed on 10 January 2023), and interproscan (https://www.ebi.ac.uk/interpro/search/sequence/) (accessed on 10 January 2023) [68,69,70]. In addition, GO, KEGG, and the Retcome pathway were inferred by using the clusterProfiler package in R [71]. The threshold was set as a *p*-value < 0.01. Hematophagous traits of a tick include host questing, blood meal digestion, detoxification of xenobiotic factors, nutrient metabolism, and an immune response. Genes related to these traits were collected, including 1148 and 1028 genes for *H. longicornis* and *R. microplus*, respectively [20].

To further explore the gene expression in *R. microplus*, transcriptomes of samples from six tissues (ovary, synganglion, salivary gland, fat body, gut) at various stages of development (embryo, partially engorged, and fully engorged female) were downloaded from the NCBI database (project ID: PRJNA232001) [49]. Diverse software programs were put to use. Trim_galore software (version 0.6.7) https://github.com/FelixKrueger/TrimGalore) (accessed on 15 April 2023) was used to filter out the low-quality reads and adapter sequences with the parameters “--length 70—quality 20—paired”. After quality control, HISAT2 (version 2.2.1) was applied so that the high-quality reads were aligned to the corresponding reference genome (ASM1333972v1) [72]. Afterwards, the read count of each gene was calculated for each sample by HTSeq (version 2.0.2) with parameters “-s no -r pos --mode=union --type exon --idattr gene_id” [73]. We used filterByExpr to filter out the low-expression genes, and calculated the counts per million (CPM) based on the trimmed mean of M-values (TMM) normalization by using the EdgeR package (version 3.38.2) [74]. To determine the gene’s differential expression in different tissues or development stages, we used GFOLD (version 1.1.4) without replicates [75]. A gene with a generalized fold change (GFOLD) value greater than or lower than zero (cutoff = 0.01) was considered differentially expressed [75]. Moreover, in order to understand the effectors that determine tick feeding activities, we downloaded the transcriptome of the tetracycline-treated *H. longicornis* nymph from the NCBI database (project ID: PRJNA 693137) and followed the same steps to process the transcriptome for subsequent analysis.

To investigate thoroughly the function of gene resistance to pathogen infection in *R. microplus* and *H. longicornis*, transcriptomes of the invertebrate pathogen *Metarhizium anisopliae* JEF-290-infected and uninfected nymph of *H. longicornis* were downloaded from the NCBI database (project ID: PRJNA714456) [76]. The high-quality reads were aligned to the corresponding reference genome (ASM1333976v2) using HISAT2 (version 2.2.1) [72]. In addition, transcriptomes of the sialotranscriptome profile of *R. microplus* in response to *Theileria equi* were downloaded from the NCBI database (project ID: PRJNA695199) [77]. The subsequent analysis followed the same detailed transcriptomic processing steps as previously described. BLASTP [78] https://blast.ncbi.nlm.nih.gov/Blast.cgi (accessed on 29 August 2023) was used to calculate the similarity between two proteins. 

## 3. Results

### 3.1. Distinct Population Structures in R. microplus and H. longicornis

**Figure 1:**
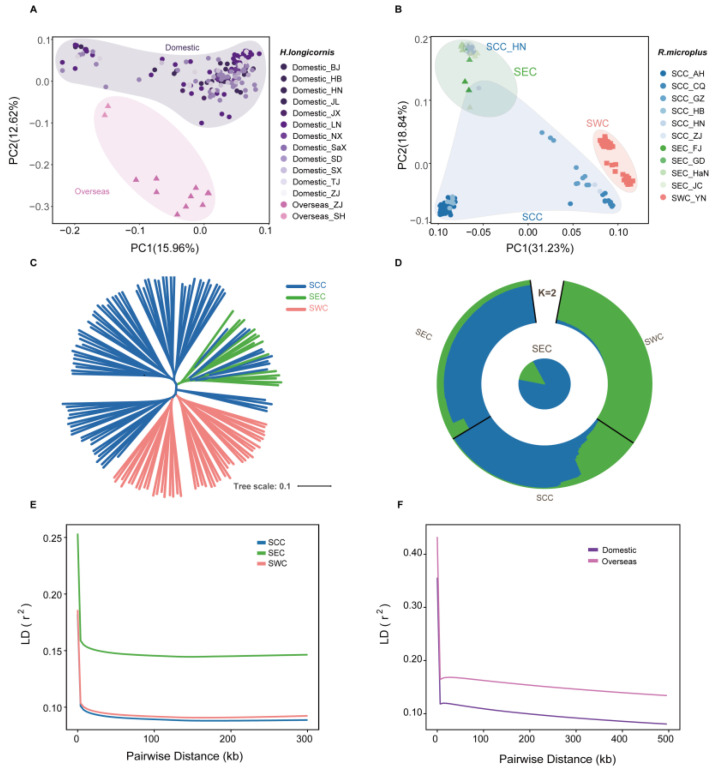
Population structures of *H. longicornis* and *R. microplus*. (**A**,**B**) Principal component analysis (PCA) plot showing segregation of *H. longicornis* and *R. microplus* individuals, respectively. Differently colored shapes represent samples from different populations and provinces. SCC, South Central China; SEC, Southeast China; SWC, Southwest China. (**C**) Phylogenetic structure of *R. microplus* populations. (**D**) Population genetic structure of all *R. microplus* sample accessions, as estimated using ADMIXTURE with the best K = 2. The outer circle graphic shows the ancestry proportions of each individual, and the center pie chart highlights the ancestry proportions of the SEC population. Each color represents a different ancestral composition. (**E**,**F**) LD decay in different populations of *H. longicornis* and *R. microplus*.

To understand the population structure and genetic analysis of *R. microplus* and *H. longicornis*, we selected available whole-genome sequencing data (mean read coverage of ~8X), including 161 *H. longicornis* from 14 regions and 140 *R. microplus* from 12 regions, for an in-depth analysis [20]. The population structure of the two tick species was characterized using a subset of autosomal variants including 177,208 variants in *H. longicornis* and 1,450,256 variants present in *R. microplus* by filtering out variants with missing rates of >10%, minor allele frequencies (MAF) of <5%, and a strong linkage disequilibrium (LD). Principal component analysis (PCA) revealed that the *H. longicornis* samples were divided into two subclades, and *R. microplus* samples were divided into three subclades in the two-dimensional PC plot (Figure 1A,B). Moreover, the phylogenetic tree implemented in IQtree (version 1.6.12) revealed the same distinct monophyletic clades for *H. longicornis* and *R. microplus* (Figure 1C and Figure S1A). Additionally, we performed ADMIXTURE analysis, which separates the study populations based on their geographic origins to determine the evolutionary history of the two tick populations by individual ancestry coefficients [57]. For *R. microplus*, the cross-validation error showed the lowest value at K = 2, regarded as the best number of ancestral populations to explain the variation for *R. microplus* (Figure 1D and Figure S1B,C). The individuals from these two sup-populations, Southeast China (SEC) and Southwest China (SWC), exhibited significantly different ancestral compositions, suggesting low gene flow due to geographic isolation. In contrast, the results for *H. longicornis* showed no substantial difference in ancestral composition between the identified clusters, which can likely be attributed to its facile invasion of new areas and prolific proliferation within established ranges, prioritizing dispersion over local competition [20].

The pair-wise LD for all high-quality SNPs was calculated to measure the genetic diversity among these subpopulations. For *R. microplus*, the LD decreased to half of its maximum value at 24 kb in SEC but was at 0.13 kb and 0.24 kb in South Central China (SCC) and SWC, respectively (Figure 1F). Specifically, the SEC population displayed a relatively slower rate of LD decay than the other populations, indicating lower genetic diversity in SEC and greater diversity in SCC. Conversely, for *H. longicornis*, the LD decay rate of domestic and overseas populations is basically the same (Figure 1E).

### 3.2. Genomic Diversity and Population Migration

**Figure 2:**
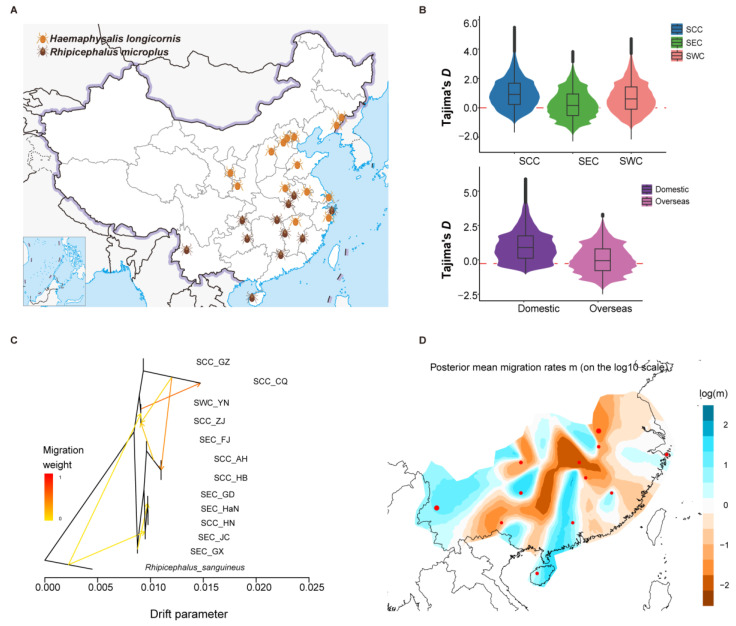
Gene diversity and gene flow of *H. longicornis* and *R. microplus* in different geographical regions. (**A**) Regional distribution of the two tick species in China. (**B**) Tajima’s *D* values of the two tick species in different regions. (**C**) Gene flow between the different regions for *R. microplus* populations. (**D**) Migratory patterns of *R. microplus* based on EEMS (estimated effective migration surface).

The geographical distributions of the two tick species in China were apparently different (Figure 2A). We estimated the nucleotide diversity (π) and Tajima’s *D* in *R. microplus* and *H. longicornis* using multiple individuals (Figure 2B and Figure S2A). For *R. microplus*, the result suggested that the lowest nucleotide diversity was in the SEC population, which is indeed lower than in the other two populations (SCC and SWC) (Figure S2A). Moreover, Tajima’s *D* showed the same result (Figure 2B). These results suggest that the SEC population significantly differed from the other two populations. The F_ST_ value measures the degree of differentiation between the two populations, with the highest value observed between the SEC and SWC populations (Figure S2A). For *H. longicornis*, Tajima’s *D* values for both populations were above zero and higher in the domestic population, suggesting potential selection pressure (Figure 2B). However, no significant difference in nucleotide diversity between domestic and overseas was observed (Figure S2B). We examined the gene flow in different regions based on TreeMix and EEMS analyses of *R. microplus* and *H. longicornis*. For *R. microplus*, the Hunan branch, located in SCC, is more similar to the SEC population, possibly due to more accessible communication facilitated by geographical proximity. In contrast, communication with the South Central branch was hindered (Figure 2C,D). Conversely, for *H. longicornis*, the EEMS results show that the migration rates of the two populations overseas are relatively low (Figure S2C,D), indicating the communication between them and the domestic population is not close. These results appropriately explain the formation of different geographical populations of the two types of ticks, providing a foundation for studying the differences in tick life history and potential virus transmission mechanisms in different regions.

### 3.3. Genetic Variations Contribute to Blood Meal Digestion and Vector–Pathogen Adaptation in R. microplus

Ingesting blood is a crucial process for tick survival and development, and the free-living stage of the hard tick *R. microplus* life cycle begins when the fully engorged female drops from the host, which involves a variety of homeostasis regulation mechanisms, including immune regulation [79]. To investigate the genomic signature responsible for the blood meal adaptation to different populations in *R. microplus*, strong genomic regions were identified using cross-population approaches (F_ST_ and XP-EHH) and one within-population approach (iHS). Accordingly, the F_ST_ calculation and XP-EHH analysis were used to detect 243 and 664 possible genomic selection regions, respectively, which overlapped 497 annotated protein-coding genes detected by at least one method mentioned above. Additionally, within-population natural selection tests identified 600 selection regions with significant (*p* < 0.05) iHS values, harboring 372 potentially selected protein-coding genes. Several genes bearing signals of positive selection in the SEC and SWC populations are associated with immune regulation in the stress response (e.g., *SNRK*, *UBE2QL1*, *CTSC*, *Vacuolar H+ ATPases*, *MAPK*, and *Hsp60*) and iron transport in the blood (e.g., Cytochrome.P450) (Table S1) [80,81,82,83,84,85,86,87,88]. Gene ontology (GO) enrichment analysis showed that the selected genes were significantly enriched in the categories related to response to hydrogen peroxide, reactive oxygen species, protein tyrosine kinase activity, cell death, and T-cell activation (Table S2), indicating that these gene categories may play critical roles in tick development, reproduction, and survival when ticks are exposed to different environments [89]. 

**Figure 3:**
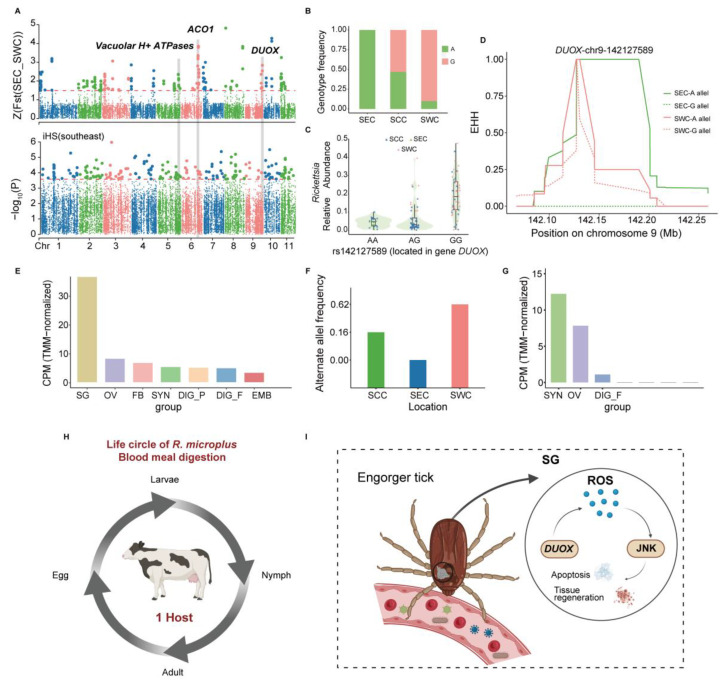
*DUOX* gene and *ACO1* gene contribution to blood digestion and the immune response of *R. microplus.* (**A**) Whole-genome scan with F_ST_ and iHS for the SNPs around *DUOX*, *ACO1,* and *Vacuolar H+ ATPases* between the Southeast China (SEC) and Southwest China (SWC) populations. F_ST_ is normalized as Z scores for *R. microplus*. The horizontal red dashed lines represent the empirical threshold for the selected regions. (**B**) Genotype frequency of rs142127589 among the three *R. microplus* populations. (**C**) Correlation of a *DUOX* SNP (rs142127589) with the abundance of *Rickettsias*. (**D**) Haplotype decay around the *DUOX*-chr9-142127589 allele in SEC and SWC populations. (**E**) Gene expression of the *DUOX* gene in different tissues or development stages. Digestive cells from fully engorged female ticks (DIG_F); fat bodies from partially and fully engorged adult females (FB); synganglion from partially and fully engorged adult females (SYN); digestive cells from partially engorged female ticks (DIG_P); embryos (EMB); ovaries from partially and fully engorged adult females (OV); salivary glands from partially and fully engorged adult females (SG). (**F**) Alternative allele frequency across the three *R. microplus* populations. (**G**) Gene expression of *ACO1* gene in different tissues or development stages. (**H**) Life circle of *R. microplus* blood meal digestion. (**I**) The *DUOX* gene contributes to blood digestion and the immune response in *R. microplus*.

We focused on the candidate gene *DUOX* because it has been identified as a robust selection signal through F_ST_ and iHS analysis in the whole genome, ranking seventh in the F_ST_ values of SEC and SWC populations (Figure 3A; Tables S1, S3 and S4). Moreover, it has been confirmed in multiple previous studies to be related to reduction–oxidation homeostasis during a blood meal in ticks. Additionally, we determined a specific site (chr9: g. 142,127,589 A > G) that ranked as the first value in the F_ST_ analysis. It showed a 0.1% maximal population branch statistic (PBS) value in the SEC population (Figure S3A), which indicated that it possibly plays an essential role in affecting and regulating the expression of *DUOX.* The variant 142,127,589 is located in the intron region of *DUOX* and may play an important role in regulating gene expression. According to our findings, the frequency of the G genotype of variant rs142127589 decreased from SEC to SCC and SWC, respectively. It was nearly absent in SEC, with the lowest alternative allele frequency of 0%, and no significant (*p* < 0.05) correlation was found between the variant rs142127589 genotype and overall pathogen abundance in different populations of *R. microplus* (Figure 3B and Figure S3B). Moreover, we observed distinct patterns in pathogen types carried by individuals with different genotypes of rs142127589, with *Rickettsias* more abundant in individuals with the G genotype (Figure 3C). Furthermore, extended haplotype homozygosity (EHH) around this SNP also indicated that the SEC population had slower homozygosity decay than other populations, confirming that the SEC population underwent a selective sweep in *DUOX* (Figure 3D). *DUOX*, a vital member of the nicotinamide adenine dinucleotide phosphate (NADPH) oxidase family, is crucial for maintaining mucosal immunity [90]. To further investigate and confirm the function of the *DUOX* gene, the transcriptomes from samples from multiple tissues at various stages of development were used for gene expression analysis, and we observed that the *DUOX* gene was highly expressed in the SGs (Figure 3E) [49]. Collectively, these findings suggest that *DUOX* plays a crucial role in immune response regulation and pathogen interactions, highlighting its potential as a key evolutionary driver of tick adaptation.

Iron homeostasis is vital for hematophagous arthropods during blood feeding, as it profoundly impacts their reproduction and development [91,92,93]. The gene LOC119172490, identified through strong selection signals in F_ST_ and XP-EHH analyses (Figure 3A and Figure S4A,B and Tables S1, S3 and S5), exhibits a higher alternative allele frequency in the SWC population compared to others (Figure 3F). We observed distinct patterns in pathogen types carried by individuals with different genotypes of rs152825916, with *Ehrlichia* less abundant in individuals with the G genotype (Figure S4C). However, no significant (*p* < 0.05) correlation was found between the variant rs152825916 genotype and overall pathogen abundance in different populations of *R. microplus* (Figure S4D). Annotated as cytoplasmic aconitate hydratase-like (*ACO1*), this gene regulates cellular iron homeostasis by acting as IRP1 in its oxidized form (Figure S4E) [94,95,96,97,98]. Transcriptome analysis revealed high *ACO1* expression in the synganglion and ovaries across developmental stages (Figure 3G). These results indicate that iron homeostasis regulation involving the *ACO1* gene plays an important role in the physiology of ticks [49].

### 3.4. Genetic Variations of H. longicornis Contribute to Heme Synthesis and Correlate with Coxiella Abundance

**Figure 4:**
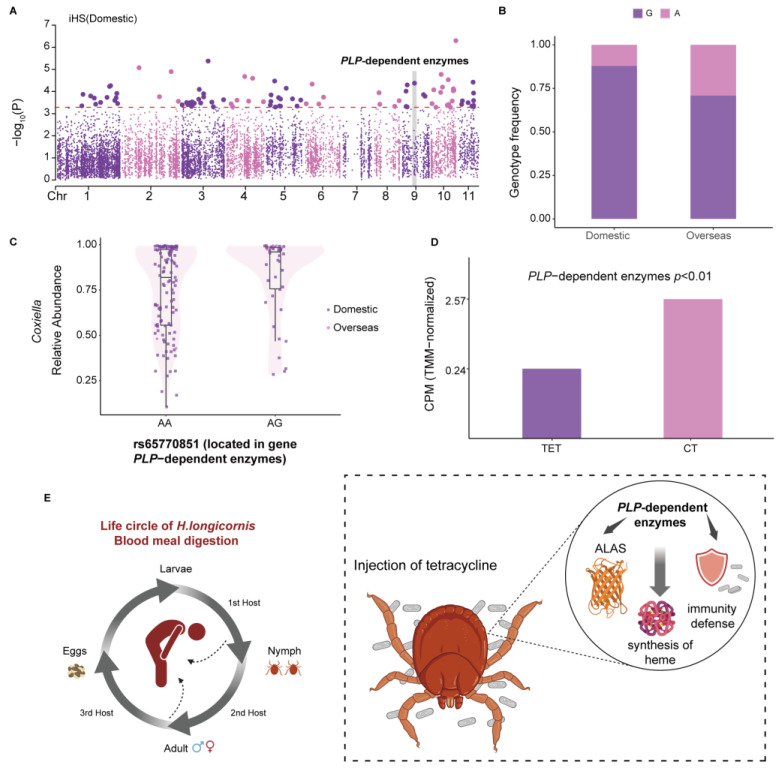
*PLP*-dependent enzyme gene contributes to blood digestion in *H. longicornis.* (**A**) Whole-genome scan with iHS: top 1% windows; the horizontal red dashed lines represent the empirical threshold for the selected regions. (**B**) Genotype frequency of rs65770851 across *H. longicornis* populations. (**C**) Correlation of a *PLP*-dependent enzyme SNP (rs65770851) with the abundance of *Coxiella*. (**D**) *PLP*-dependent enzymes in nymphs treated with tetracycline (TET) versus controls (CT) (**E**) *PLP*-dependent enzyme genes contributing to blood digestion and heme synthesis in *H. longicornis*.

*H. longicornis*, which is widely distributed and predominant in at least 17 provinces in China, has a complex genetic landscape that is closely linked to its geographic distribution [99]. It is critical to understand its genetic complexity and determine the links between its genomic variation and geographic distribution. To elucidate a potential genetic candidate for these differences, we used two cross-population approaches (F_ST_ and XP-EHH) and one within-population approach (iHS) to scan the genomic selective region between domestic and overseas populations. Notably, our F_ST_ and XP-EHH results did not show significant selection signals. We focused on the domestic population and identified 29 candidate genes detectable by the iHS (Table S6). The KEGG enrichment analysis revealed that the selected genes were significantly (*p* < 0.05) enriched in the categories related to amino acid synthesis and metabolism (Table S7). The HaeL19522 gene was particularly of interest, with notable positive selection in the domestic population that ranked fourth, and it showed remarkable enrichment in the KEGG pathway (Figure 4A; Table S7). Moreover, this gene showed a noticeable difference in genotype frequency between domestic and overseas populations, with the AA genotype carrying a wider variety and abundance of pathogens in the domestic population (Figure 4B and Figure S5A). Interestingly, for variant rs65770851, the pathogen *Coxiella* exhibited a unique abundance pattern, which was more prevalent in individuals with the G genotype in both populations (Figure 4C). 

We annotated the HaeL19522 gene as a *PLP*-dependent enzyme through protein homologous analysis. *PLP*-dependent enzymes refer to a class of enzymes that rely on the coenzyme pyridoxal phosphate (*PLP*, also known as the active form of vitamin B6), which participate in many vital biochemical reactions in organisms, covering a wide range of metabolic pathways and biological functions [100,101]. To investigate the function of this gene in tick blood meal digestion, we compared gene expression data between *H. longicornis* nymphs treated with and without tetracycline. Treatment with tetracycline impacts the blood feeding of ticks, mainly by reducing the number of endosymbionts, such as *Coxiella,* which can promote tick blood feeding [102]. The expression of *PLP*-dependent enzymes was markedly reduced after tetracycline treatment, indicating that *PLP*-dependent enzymes play an essential role in blood feeding (Figure 4D,E). We also investigated the gene expression between the invertebrate pathogen M. anisopliae JEF-290-infected and uninfected ticks. *PLP*-dependent enzymes revealed a slightly lower expression in uninfected ticks compared with infected ones (|generalized fold change/GFOLD| = 0.584128 > 0.01) (Figure S5B). M. anisopliae JEF-290 significantly controls tick transmission, indicating that *PLP*-dependent enzymes have a particular role in tick immunity. The above results showed that the *PLP*-dependent enzymes/HaeL19522 gene are meaningful for *H. longicornis* during blood feeding and its immunological defense against pathogens.

### 3.5. Correlation Between Genetic Variants of Ticks and Tick-Borne Pathogen Abundance

Understanding the interaction between pathogens and ticks is essential for elucidating the mechanisms of TBDs. We hypothesized that highly differentiated SNPs between tick populations may likely to influence the differentiated pathogens in ticks. Thus, we investigated the correlation between different tick genetic variates and microbiome species that were differentially prevalent across tick populations. In total, 28,133 filtered SNPs in *R. microplus* (see Section 2), 4229 in *H. longicornis*, and 12 filtered microbiome species (see Section 2) were selected for correlation analysis. Among these features, we identified significant correlations of SNPs with species using a meta-analysis of different populations in these two tick species. Our meta-analysis revealed no significant SNPs associated with pathogens in *H. longicornis*. In contrast, we identified several SNPs in functional genes related to blood feeding in *R. microplus*, suggesting significant differences in the association of pathogens with these two tick species (Table S12).

**Figure 5:**
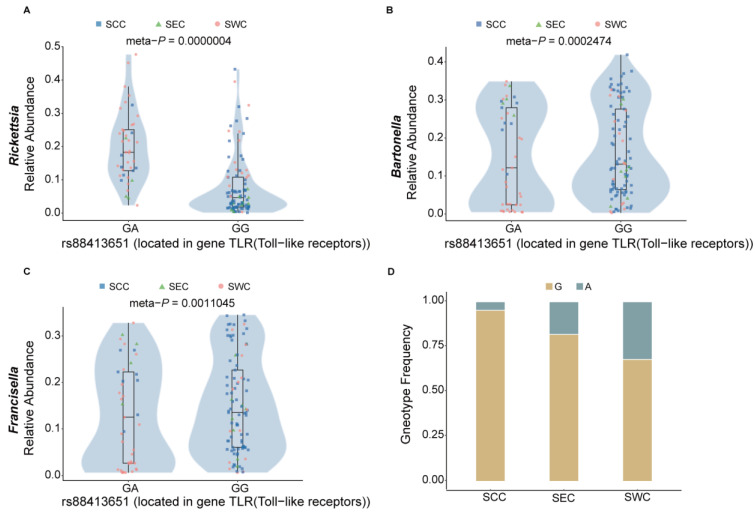
The tick genetic variant rs88416395 is correlated with the abundance of tick-borne pathogens. (**A**–**C**) Correlation of a Toll-like receptors SNP (rs88418395) with *Rickettsia, Bartonella,* and *Francisella* abundance, respectively. (**D**) Genotype frequency of rs88418395 among the SCC, SEC, and SWC populations.

We found that a certain number of species and their correlated tick SNPs could affect tick immunity. For instance, the species *Rickettsia* was observed to be the most differentiated species between SCC and SWC (*p* = 9.48 × 10^−10^, Wilcox test; Table S9) and between SEC and SWC (*p* = 1.37 × 10^−6^; Wilcox test; Table S10). *Rickettsia* infection may trigger a series of innate immune responses in ticks, including three main immune signaling pathways of arthropods: Toll, IMD, and JAK/STAT [103]. Its correlated SNP (rs88416395, meta-*p* = 4 × 10^−7^, Spearman correlation test) is located in the immune-related gene TLR, which is a primary sensor of microbial pathogens that activates innate immune cells and initiates and orchestrates adaptive immune responses [104]. The correlation pattern of this locus with *Rickettsia* is opposite to that of *Bartonella* and *Francisella* (Figure 5A–C), indicating that different microbial species may induce distinct immune patterns, potentially influencing pathogen–host interactions, and this phenomenon that has been widely observed in other arthropod species as well [105]. Additionally, in the southwest region, the GA genotype frequency is higher than in the other two regions (Figure 5D), indicating different patterns of association between microbes and regions. This suggests the presence of specific microbial species in a given geographic region may exert selective pressure on the tick population, favoring certain genotypes that confer better resistance or tolerance to local pathogens. 

Moreover, the species *Francisella* has been reported to have successfully transitioned from mammalian to arthropod hosts by sensing changes in iron and/or altering the expression of iron-regulated genes in ticks [106]. Its associated SNP (rs210552537, meta-*p* = 1.91 × 10^−3^) is located in the UTR3 of protein-coding gene cytochrome P450 reductase, which is an essential redox partner for all cytochrome P450 enzymes, facilitating the transfer of electrons from NADPH into numerous physiological reactions [107]. The cytochrome P450 reductase complex, in conjunction with the iron component poly(rC)-binding protein 2, forms an auxiliary system that assists in heme catabolism and iron transfer [108]. Iron-regulated genes have been shown to play an essential role in regulating the virulence of *Borrelia burgdorferi* [109]. Under iron-limiting conditions, many differentially expressed genes of LVS of *F. tularensis* are related to virulence or intracellular replication. Our data showed that the frequency of the G genotype of the variant rs210552537 decreases from SCC to SEC and SWC populations (Figure S6A,B) [110]. This pattern of decreasing G allele frequency could indicate a selective pressure exerted by the regional differences in microbial communities, with implications for the ability of *Francisella* to adapt and survive in varying ecological niches.

## 4. Discussion

The differences between *H. longicornis* and *R. microplus* are significant in terms of their ecological roles, host interactions, and implications for disease transmission [111]. The geographical distribution and population interaction of *H. longicornis* and *R. microplus* exhibit strikingly different patterns, reflecting distinct ecological and biological traits between these two tick species, which is consistent with previous findings based on SNV and mtDNA data [20,112]. Building upon genetic structure analysis, we identified two distinct branches within *H. longicornis*, comprising domestic and overseas populations. In contrast, the geographical distribution pattern of *R. microplus* was more clearly delineated, with three branches, the south central, southeast, and southwest, mirroring previous population structure inferences [20]. Furthermore, population migration and gene flow analyses highlighted that gene flow between populations of *R. microplus* was more extensive compared to *H. longicornis*. The divergence in population structure and interaction can be attributed to differences in survival strategies, ecological niches, and life cycle characteristics [113]. *H. longicornis* displays remarkable adaptability, thriving across diverse habitats such as grasslands and forest edges in temperate regions, which supports its widespread distribution [114,115]. Its three-host life cycle facilitates rapid dispersal and reduces genetic differentiation among populations [20]. In addition, *H. longicornis* has the unique feature of having both parthenogenetic and bisexual populations [116]. Moreover, studies have shown that parthenogenetic populations spread more rapidly than bisexual ones, and phylogenetic analysis revealed distinct lineages for these populations, suggesting no gene flow between them [112,117]. However, this relationship could not be explored in the present study due to the lack of data on the absence of an assembled genome for parthenogenetic *H. longicornis*. In contrast, *R. microplus* is predominantly confined to warm, humid environments, typically in tropical and subtropical regions, where its distribution is closely tied to livestock farming [118]. This single-host species feeds primarily on cattle, and the regional differentiation of its populations may be influenced by variations in cattle breeds and associated farming practices across different regions [119,120]. The interplay of ecological and biological factors highlights the critical influence of species-specific traits, including life cycle strategies, host specialization, and environmental adaptability, in shaping the population structure and geographical distribution of tick species [121]. These findings underscore the importance of comprehensive genomic studies to uncover the genetic and evolutionary mechanisms driving tick ecology and to enhance our understanding of their roles in disease transmission and host interaction.

Differentiated habitats and species-specific traits are critical in shaping the genomic adaptations and evolutionary mechanisms of the two tick species, *H. longicornis* and *R. microplus*. Both species demonstrate genomic adaptations associated with blood feeding and coevolution with pathogens, yet they exhibit notable differences in the specificity of their adaptive evolution. For *H. longicornis*, statistical analyses across populations identified genes involved in heme synthesis, particularly *PLP*-dependent enzymes. *PLP*-dependent enzymes may play an essential role in heme synthesis. Previous research has reported that ALAS is a type of *PLP*-dependent enzyme, which is the starting enzyme of the heme synthesis pathway and catalyzes the reaction between histidine and pyruvate to form δ-aminoketoglutaric acid [122,123]. *PLP*-dependent enzymes exhibit significant genotype and allele frequency differences between domestic and overseas populations, reflecting distinct ecological adaptations. Furthermore, the genotype frequencies of *PLP*-dependent enzymes correlate positively with the abundance of *Coxiella*. Previous studies have shown that endosymbiont *Coxiella* is vital for regulating tick 5-HT biosynthesis and feeding [102]. Experimental treatment with tetracycline significantly reduced *Coxiella* abundance in *H. longicornis* and concomitantly decreased the expression levels of *PLP*-dependent enzyme genes, suggesting their critical role in reproductive development during blood feeding. In contrast, *R. microplus* displays a stronger association between its genomic features and host-specific adaptations. Genes critical for blood digestion and immune responses, such as LOC119178146/*DUOX*, have been identified. *DUOX*, an oxidative stress enzyme essential for mucosal immunity, plays a central role in tick–pathogen interactions, disease transmission, and blood meal processing [90]. In *Drosophila*, previous studies have shown that *DUOX* helps maintain intestinal redox homeostasis in the midgut [124]. In *A. gambiae*, the heme immunomodulatory peroxidase (IMPer), secreted by its intestinal epithelial cells, catalyzes the formation of a non-cellular molecular barrier called a dityrosine network (DTN), which blocks the transmission of epithelial immune elicitors and plays a vital role in intestinal epithelial cell immunity and maintaining host–microbial homeostasis [125]. Previous studies have shown that the DTN also exists in *I. scapularis* ticks [126]. RNAi-mediated *DUOX* knockdown reduces levels of *B. burgdorferi* persistence in ticks, with the absence of DTN formation [126]. Transcriptomic analysis revealed that the *DUOX* gene is highly expressed in the salivary glands of ticks [49]. Tick SGs are organs of osmoregulation in ticks. Both the SGs and saliva have been investigated mainly for their indispensable role in the response to pathogen infection and transmission, host hemostasis, and immune responses [127,128,129,130]. Previous studies have shown the same result, that a transcript annotated as *DUOX* A has been suggested to act in the trafficking of *DUOX* to the cell plasma membrane in mammalian liver, which has shown higher transcriptional levels in SGs from fully engorged adult female ticks [131]. Moreover, in the salivary glands of ticks, many signals related to degeneration and the onset of apoptosis have been described [132]. *DUOX* plays a key role in the JNK pathway. During the early stages of apoptosis in tick salivary gland cells, NADPH oxidase mediates the formation of ROS and activates the c-Jun N-terminal kinase (JNK) pathway, which in turn activates the *moladietz* (*mol*) gene [133,134]. JNK transcriptionally activates the gene that encodes the *DUOX* maturation factor (NPI), which is essential for ROS production, and in turn, triggers JNK activation during tissue regeneration, creating a feedback loop that maintains JNK activity throughout the regeneration process (Figure 3I) [131,133,134]. Furthermore, the enzyme produced by *DUOX* can directly damage pathogens by generating reactive oxygen species, produce antimicrobial peptides such as Duox/Mesh in the Lmd and JAK/STAT signaling pathways, mediate ROS expression, and trigger melanosis cascades [135]. This pathway involves various bactericidal substances and also plays a critical role in intestinal immunity [134]. Furthermore, the genotype frequencies of rs142127589 loci within *DUOX* are highly correlated with pathogen abundance and exhibit pronounced regional variation, aligning with tick ecological adaptations and pathogen transmission dynamics. Previous studies have reported that infection with *Rickettsia parkeri* affects the physiology and gene expression of *Amblyoma maculatum* ticks [136]. Thus, the frequency of the G genotype of the variant rs142127589 and its correlated *Rickettsias* might be one of the factors affecting the role of *DUOX* in different regions. Additionally, *R. microplus* demonstrates genomic adaptations linked to iron transport during blood-feeding. The gene LOC119172490/*ACO1*, encoding a key enzyme in iron and ROS metabolism, is highly expressed in the ganglia and ovaries, indicating its role in reproduction, development, and pathogen transmission [49,96]. Previous studies have confirmed that silencing IRP1-related genes, including *ACO1*, has been shown to reduce female hatching rates and post-blood-meal weights [137]. Notably, variation allele frequencies (VAF) for *DUOX* and *ACO1* across the three *R. microplus* populations align with regional cattle breed distribution. Resistant cattle breeds predominate in the southern regions (lower VAF), while hybrid tick-resistant and susceptible breeds dominate the central and southwestern regions (higher VAF). This host specificity and regional variation underscore the coevolution of *R. microplus* with Asian cattle, as previously documented [138]. Together, these findings highlight both shared and divergent genomic adaptations in *H. longicornis* and *R. microplus*. While both species exhibit coevolutionary traits with pathogens and blood-feeding adaptations, *R. microplus* shows a stronger link between its genomic features and host-specific ecological variations, reflecting distinct pathways of adaptive evolution.

Ticks and pathogens interact in complex ways that significantly influence the vectorial competence of ticks, playing a key role in TBDs [139]. Pathogens transmitted by ticks have evolved mechanisms to survive in their arthropod hosts by manipulating gene expression in specific environments like the midgut or salivary glands [140,141]. These interactions can impact various aspects of tick biology, including nutrition, fitness, development, reproduction, immune response, and tolerance to environmental stress [142]. The ecological and biological traits of different tick species can shape these interactions and influence their capacity to transmit pathogens [143]. *H. longicornis* exhibits a broad host range, infesting various mammals, including livestock, wildlife, and humans, which contributes to its potential as a vector for multiple pathogens, such as *Borrelia* spp. and *Anaplasma* spp. [1]. This versatility allows *H. longicornis* to adapt to diverse ecological niches, making it a notable concern for zoonotic diseases. In contrast, *R. microplus* is more specialized, primarily targeting cattle, and is responsible for transmitting significant pathogens such as *Babesia bovis* and *Anaplasma marginale* [144]. Life cycle dynamics further distinguish these ticks. *H. longicornis* follows a three-host life cycle, where each developmental stage (larva, nymph, adult) typically feeds on a different host [114]. This multi-host requirement can enhance its ability to spread pathogens across species [145]. In contrast, *R. microplus* is capable of completing its life cycle on a single host, allowing for increased reproduction rates and more efficient establishment in cattle populations [146]. This trait not only facilitates its persistence in endemic regions but also enhances its role in pathogen amplification [147]. Our analyses of pathogen abundance in the two tick species revealed further distinctions. We quantified the abundance of 12 pathogens and performed association studies between normalized pathogen loads and positively selected loci. In *H. longicornis*, no significant associations were identified. However, in *R. microplus*, we found two significant SNPs (rs88416395, meta-*p* = 4 × 10^−7^; rs210552537, meta-*p* = 1.91 × 10^−3^) that were strongly associated with the microbes *Rickettsia* and *Francisella*, and further studies have shown that the two genes are related to the immune system and iron metabolism of ticks (Table S12) [104,107]. Previous studies have shown that *Rickettsia* and *Francisella*, commonly found in species like *R. microplus*, are known to affect tick reproductive success and development by correlating with diverse pathogenic mechanisms subverting host immunity [148]. They may also enhance vector competence by directly interacting with host immune genes to balance pathogen clearance and microbial tolerance [148]. We further found through analysis that the three regions of *R. microplus* distribution had different genotype association patterns with *Rickettsia* and *Francisella*. The relationship between genotype and pathogen diversity also suggests that specific genetic variants may be selectively advantageous in certain regions, especially in the face of regionally distinct microbial communities [149]. In addition, we also observed that the genes involved in these two loci mentioned before were related to the immune defenses of tick, which further indicated the specific targeting of the immune adaptation of the genome under the ticks in different regions with different geographical habitats [20,111]. Moreover, these differences in pathogen associations between the two tick species may stem from their divergent life history traits. The one-host life cycle of *R. microplus* creates a stable host environment that supports the accumulation and retention of specific symbionts or pathogens, such as *Rickettsia* and *Francisella*. This stability may enhance its vector competence for certain pathogens [150]. In contrast, the multi-host life cycle of *H. longicornis* exposes it to diverse microbiomes and environmental factors across hosts, potentially leading to a broader but less stable pathogen community [34]. Furthermore, these interactions between ticks and pathogens reveal how pathogens activate and manipulate the biological responses of ticks to promote survival, while the tick must balance pathogen restriction with maintaining fitness and vector competence [103,151]. These results suggest that *R. microplus* may have evolved genetic adaptations that affect its ability to host and transmit certain pathogens, providing new insights into the molecular drivers of vector competence. 

In summary, this study provides a comprehensive genomic perspective on the genetic diversity and variations within *H. longicornis* and *R. microplus*. We identified the gene HaeL19522 as a *PLP*-dependent enzyme associated with heme synthesis in *H. longicornis*. In *R. microplus*, during the blood-feeding process, we discovered two important immune-related genes, LOC119178146/*DUOX*, with the G allele frequency of rs142127589 showing a highly positive correlation with *Rickettsia* abundance. Additionally, we identified LOC119172490/*ACO1*, a gene related to iron transport. Interestingly, unlike the *DUOX* gene, the G allele frequency of rs152825916 in this gene displayed a negative correlation with *Ehrlichia* abundance. We also identified distinct patterns of pathogen associations in *H. longicornis* and *R. microplus*. Notably, we found two pathogens—*Rickettsia* and *Francisella*—that are significantly associated with *R. microplus*, playing crucial roles in immune response and iron metabolism, respectively. These findings enhance our understanding of the genomic adaptations and evolutionary mechanisms in both tick species, illuminating how specific genetic traits may facilitate pathogen transmission. Furthermore, our analysis strengthens the conceptual framework regarding the interactions between tick genetics and pathogen dynamics, highlighting potential molecular targets for controlling TBDs. By understanding these relationships, we can develop more effective strategies for managing tick populations and reducing the risk of pathogen transmission.

## Data Availability

The whole-genome re-sequencing data of 177 *H. longicornis* and 151 *R. microplus* were downloaded from the NGDC database (project ID: PRJCA002242). The reference genomes ASM1333976v2 (*H. longicornis*) and ASM1333972v1 (*R. microplus*) were download from NGDC (project ID: PRJCA002240) or NCBI (project ID: PRJNA633311). The transcriptome data of two *H. longicornis* and seven *R. microplus* were downloaded from NCBI (project ID: PRJNA714456 and PRJNA232001, respectively).

## Supplementary Materials

The following supporting information can be downloaded at: https://www.mdpi.com/article/10.3390/pathogens14040306/s1.
