# Supplementary material for: Genomic Insights into Vector–Pathogen Adaptation in Haemaphysalis longicornis and Rhipicephalus microplus
Source: Pathogens. 2025 Mar 23;14(4):306. doi: 10.3390/pathogens14040306 (PMC12030188; doi:10.3390/pathogens14040306)
Supplement: Supplementary file 1 [file pathogens-14-00306-s001.zip › Supplyment figure.pdf]

Supplementary Figures

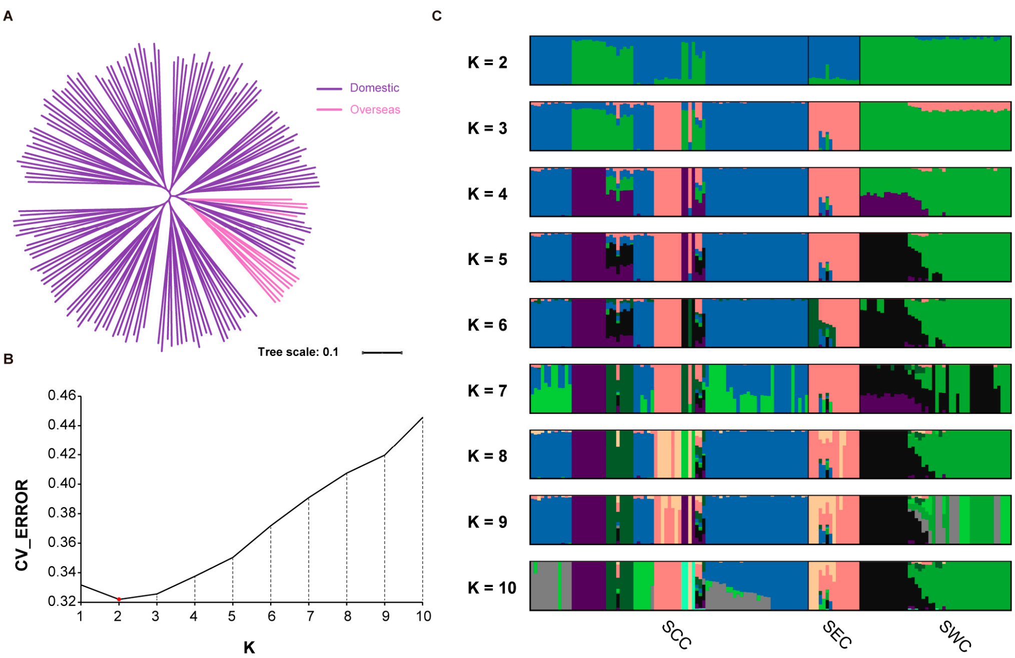

Figure S1. Population structure of ticks.

(**A**): Phylogenetic structure of *H. longicornis* populations. (**B)**: Estimation of the best K by the CV error, inferred with the ADMIXTURE program. The best K is highlighted with red dot. (**C)**: Population structure of all *R. microplus* accession estimated by ADMIXTURE with K=2-10. Each color represents one ancestral component. The x-axis represents the different accessions, and the y-axis quantifies the proportion of inferred ancestral lineages.

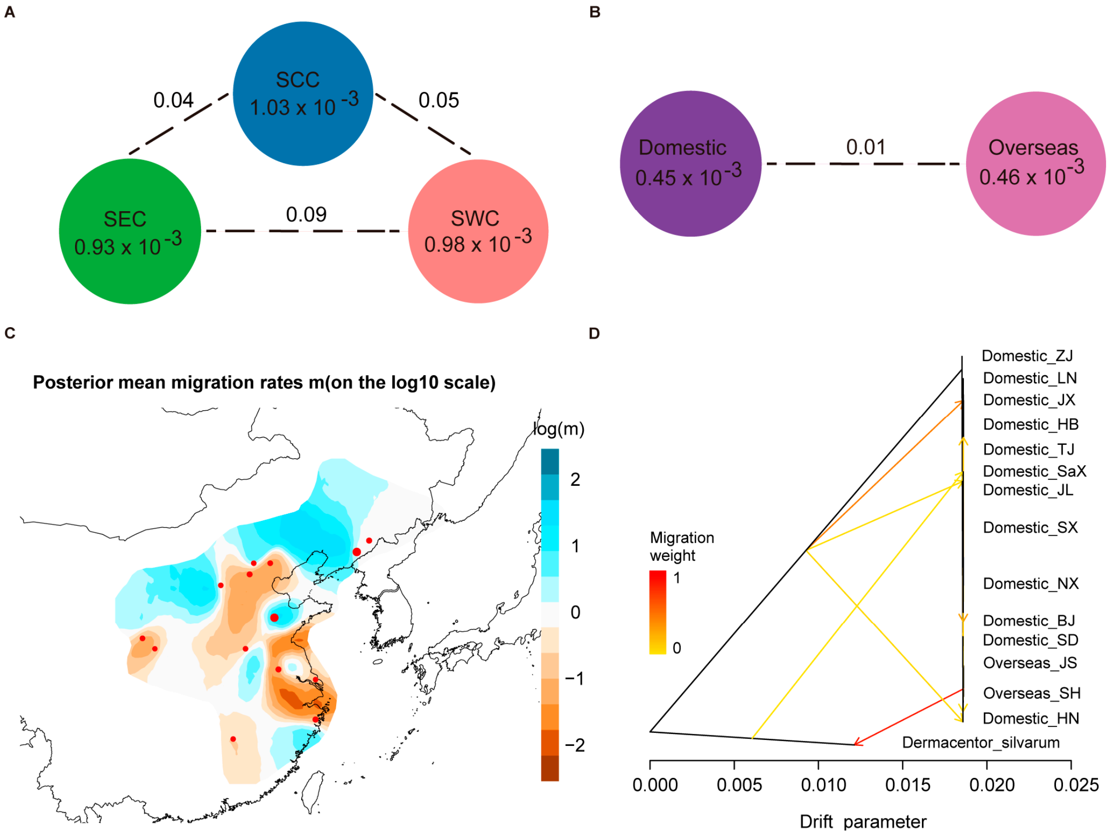

Figure S2. Gene Diversity and Gene Flow of Two Species of Ticks in Different Geographical Regions.

(**A**): Nucleotide diversity in different populations and Pairwise F_{ST} values between population of *R. microplus*. (**B**): Nucleotide diversity in different populations and Pairwise F_{ST} values between population of *H. longicornis*. (**C**): Migratory patterns of *H. longicornis* based on EEMS. (**D**): Gene flow between different regions of *H. longicornis* populations.

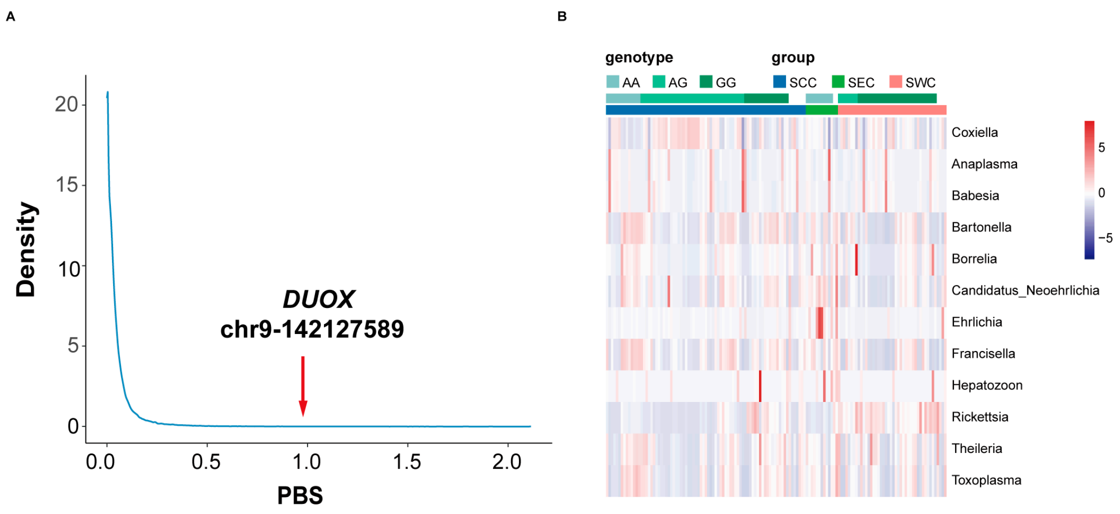

Figure S3. Genome-wide selective scanning with F_{ST} methods between SEC and SWC in *R. microplus*.

(**A**): Kernel density distribution of F_{ST}-based PBS statistics for the SEC and SWC branches. A SNP site (chr9-142127589 A > G) with top 0.1% PBS value is labeled. (**B**): The correlation heatmap between the abundance of known tick-borne pathogens and the genotype at site (chr9-142127589 A > G) in different populations of *R. microplus*.

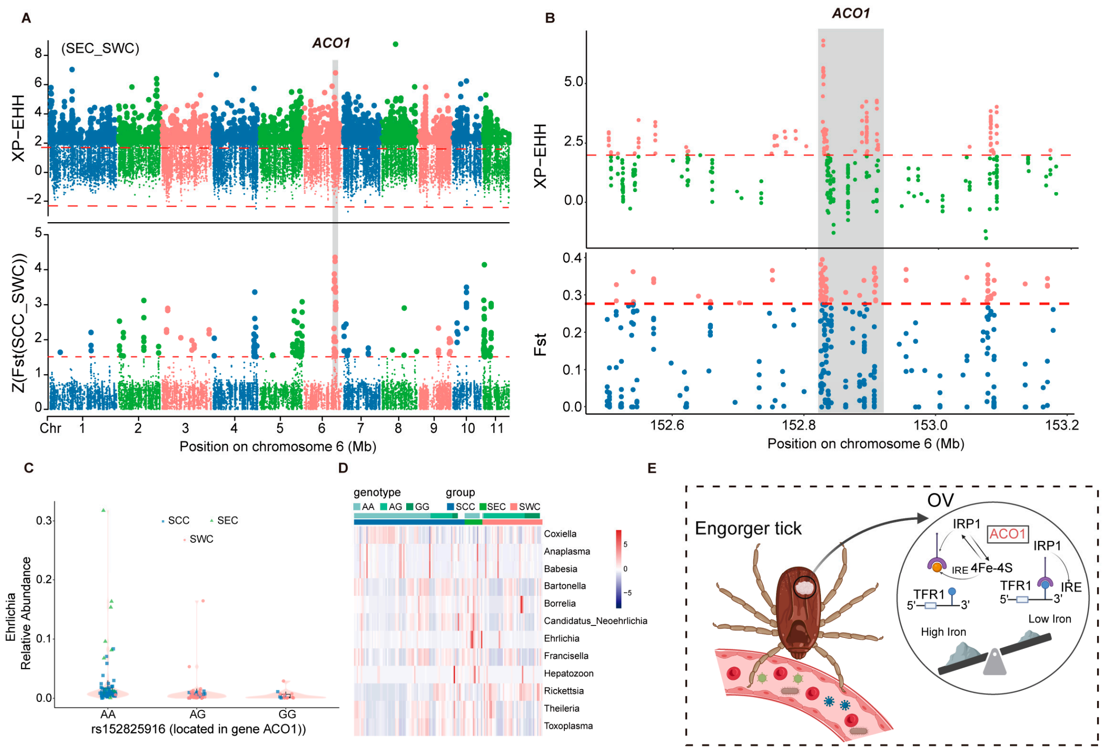

Figure S4. ***ACO1*** **gene contribution to blood digestion of** ***R. microplus.***

(**A**): Whole-genome scan with F_{ST} and XP-EHH. XP-EHH: top 1% windows; F_{ST} is normalized as Z scores for *R. microplus*. The horizontal red dashed lines represent the empirical threshold for the selected regions. (**B**): XP-EHH and F_{ST} for the SNPs around *ACO1* on chromosome 6. (**C**): The correlation of a *ACO1* SNP (rs152825916) with the abundance of *Ehrlichia*. (**D**): The correlation heatmap between the abundance of known tick-borne pathogens and the genotype at site (chr6-152825916 A > G) in different populations of *R. microplus*. (**E**): The *ACO1* gene contributes to blood digestion and the control of iron homeostasis in *R. microplus*.

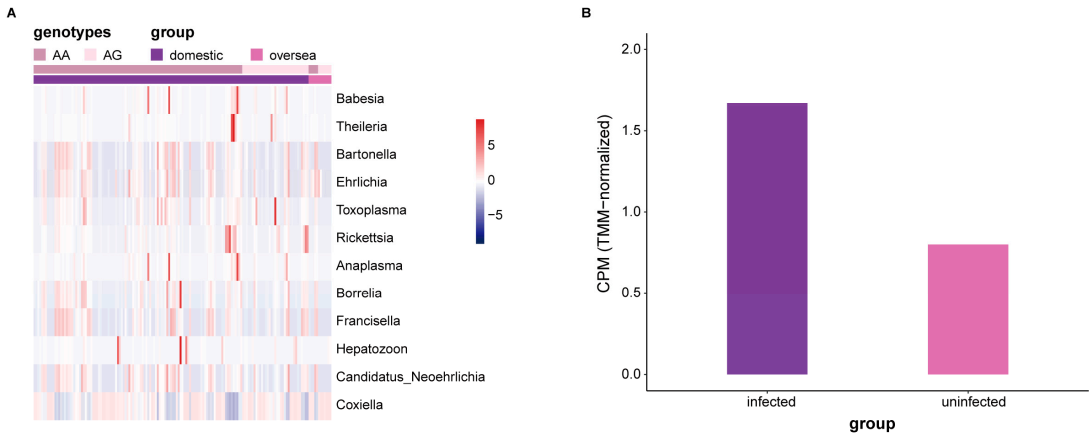

Figure S5. Relationship between the genotype at site (chr9-rs65770851 A > G) and pathogens abundance in different populations in *H. longicornis and PLP*-dependent enzymes gene contribution to vector-pathogen adaptation of *H. longicornis*.

(**A**): The correlation heatmap between the abundance of known tick-borne pathogens and the genotype at site (chr9-rs65770851 A > G) in different populations of *H. longicornis*. (**B**): Gene expression of *PLP*-dependent enzymes gene in Metarhizium anisopliae JEF-290-infected and uninfected ticks.

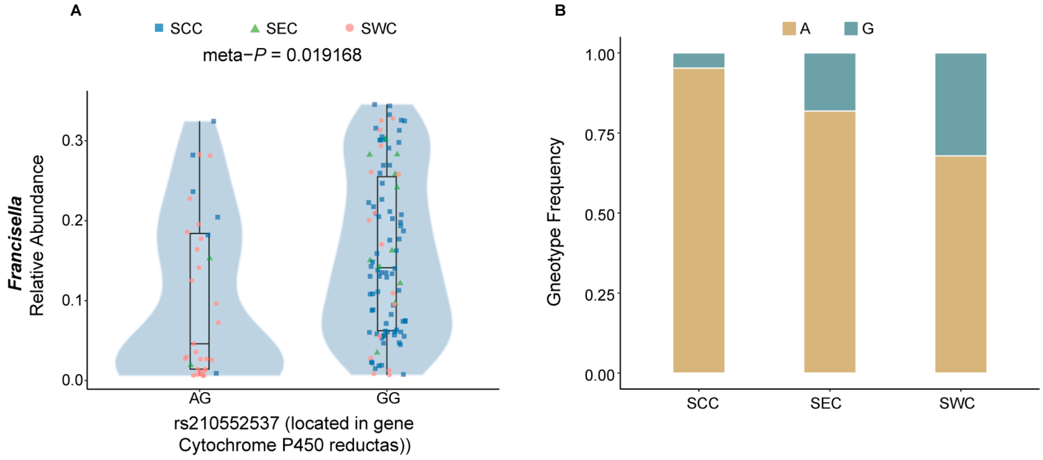

Figure S6. The Tick genetic variant rs210552537 was correlated with the abundance of *Francisella*. (**A**): The correlation of a Cytochrome P450 reductase SNP (rs88418395) with the abundance of *Francisella*. (**B**): the genotype frequency of rs88418395 among the SCC, SEC and SWC populations.

Supplementary Table S1. Identification of immune regulatory and iron transport in the blood genes with positive selection signals in SEC and SWC populations.

Chr	Start	End	Gene	Gene function	Method/Rank
8	160485001	160500000	*SNRK*	May play a role in hematopoietic cell proliferation or differentiation[80].	F_{ST}/3; XP-EHH/5
9	152390001	152405000	*DUOX*	Dual oxidase are plasma. membrane-targeted hydrogen peroxide generators which plays a vital role in support extracellular hemoperoxidases [81].	F_{ST}/7; iHS/36
6	153070001	153085000	*Aconitase*	Aconitase is one of the Fe–S proteins which plays a vital role in redox sensing and signaling reactions[82].	F_{ST}/9; XP-EHH/2
8	67260001	67270001	*UBE2QL1*	It enables ubiquitin conjugating enzyme activity [83].	XP-EHH/1
4	16170001	16180001	*CTSC*	It Encodes a member of the peptidase C1 family and lysosomal cysteine proteinase in immune system[84].	XP-EHH/3
5	190550001	190565000	*Vacuolar H+ ATPases*	It is a highly conserved evolutionarily ancient enzyme with remarkably diverse functions in eukaryotic organisms [85].	F_{ST}/15
11	5885001	5900000	*MAPK*	This gene encodes a member of the MAP kinase family that involved in directing cellular responses to a diverse array of stimuli [86].	F_{ST}/33
7	82660001	82670001	*Cytochrome.P450*	Cytochromes P450 are a superfamily of enzymes that plays roles in iron metabolism [87].	XP-EHH/37
9	148550001	148565000	*Hsp60*	HSP60 is a family of heat shock proteins that plays a vital role in prevent misfolding of proteins during stressful situation [88].	F_{ST}/44
